# Observations on rift valley fever virus and vaccines in Egypt

**DOI:** 10.1186/1743-422X-8-532

**Published:** 2011-12-12

**Authors:** Samia Ahmed Kamal

**Affiliations:** 1 Virology department, Animal Health Research Institute, Dokki, Giza, Egypt

**Keywords:** Rift Valley fever virus, RVFV, Inactivated RVF vaccines, MP12, Live attenuated RVF vaccines, Smithburn strain.

## Abstract

Rift Valley Fever virus (RVFV, genus: Phlebovirus, family: Bunyaviridae), is an arbovirus which causes significant morbidity and mortality in animals and humans. RVFV was introduced for the first time in Egypt in 1977. In endemic areas, the insect vector control and vaccination is considering appropriate measures if applied properly and the used vaccine is completely safe and the vaccination programs cover all the susceptible animals. Egypt is importing livestock and camels from the African Horn & the Sudan for human consumption. The imported livestock and camels were usually not vaccinated against RVFV. But in rare occasions, the imported livestock were vaccinated but with unknown date of vaccination and the unvaccinated control contacts were unavailable for laboratory investigations. Also, large number of the imported livestock and camels are often escaped slaughtering for breeding which led to the spread of new strains of FMD and the introduction of RVFV from the enzootic African countries. This article provide general picture about the present situation of RVFV in Egypt to help in controlling this important disease.

## Introduction

Rift Valley fever virus (RVFV, genus *Phlebovirus *and family *Bunyaviridae*), was isolated from infected sheep in 1930 [1,2]. RVFV is an arbovirus that can be transmitted directly (between vertebrates during the manipulation of infected tissues, and between mosquitoes by vertical transmission) and indirectly (from one vertebrate to another by mosquito-borne transmission) [3,4]. It is enveloped, segmented into three parts, single stranded RNA virus. The three segments are; small (S), medium (M) and large (L). The S segment is of ambisense polarity and encodes for two proteins; nucleocapsid protein (N) that coats the viral genome and a nonstructural protein (NSs). The NSs is a filamentous nuclear protein, expressed by a virus that replicates and assembles in the cytoplasm of infected cells. The NSs protein has been identified as a major virulence factor. The phosphoprotein NSs is not essential to viral replication in tissue culture thus allowing clones carrying deletions in NSs to predominate as they replicate more rapidly [4]. The M segment encodes for two proteins (NSm) of 14 kDa and 78 kDa and envelope glycoproteins (G1 and G2) which play an important role in RVFV infection and pathogenesis [2]. The L segment encodes the viral RNA-dependent RNA polymerase [5-8].

RVFV is a dose dependant pathogen. Whenever, the dose of RVFV was decreased, the onset of the disease and the time of death were delayed [9]. RVFV has only one serotype [10], but strains exist of variable virulence [11]. This genetic stability is assumed to result from a fitness trade-off imposed by host alternation, which constrains arbovirus genome evolution. Otherwise, no genetic changes were found in viruses that were passaged alternately between arthropod and vertebrate cells. Furthermore, alternating passaged viruses presenting complete NSs gene remained virulent after 30 passages. Therefore, alternating replication is necessary to maintain the virulence factor carried by the NSs phosphoprotein [4].

RVFV causes a significant threat to both human and animal health [12]. It was recorded that during periods in which human epidemics arise they are preceded by epizootics in livestock. These livestock epizootics serve as an amplification step in the spread of the virus. Prevention of disease in animals through the use of safe and effective vaccine would serve to prevent human disease by breaking the amplification cycle [13].

## The endemic status of RVFV in Egypt

According to the facts that most arthropod-borne viruses (arboviruses) are RNA viruses, which are maintained in nature by replication cycles that alternate between arthropod and vertebrate hosts [4]. RVFV ability to persist in nature depends upon certain factors which are present in Egypt [14]. These factors are: 1- the presence of unvaccinated susceptible livestock, camels and wild animals in large number over large areas, would play the major role in the ability of RVFV to establish the endemic cycle [15,16], 2- the presence of suitable environmental conditions for the insect vector propagation in the absence of effective vector control programs. 3- The vaccination with RVF live attenuated vaccines (Smithburn's strains) plays important role in the persistence of this endemicity in Egypt because it contaminate the environment and transmitted by insect vector [17]. 4- The vaccination programs were alternating between the killed and the live vaccines, the latter was used before, during or after the outbreaks in unidentified manner. 5- The vaccination by the inactivated vaccines is not covering all the susceptible animals. 6- The human losses in the first RVFV in 1977 were massive as a result of the lack or absence of public health education beside the delay in announcing the problem and delay of control programs. However, these social and medical situations are not changed till the present time. 7- The geographical continuity with Africa and the Sudan, and the Nile River facilitate the continuous introduction of infected livestock, camel or wild animals. 8- The importation of livestock from the horn of Africa and the Sudan which are enzootic countries. The government of Egypt instructions for importing live animals was to slaughter them upon arrival. Contrarily, these importing animals are usually mixed with the native breeds and moving freely in different localities of the country. 8- The field trials performed by different scientific institutions in Egypt are not under control, and participate in the environmental contaminations with the live vaccinal strains.

## Inter-epizootic periods

The scientific data about the inter-epizootic periods of RVFV in Egypt are missed. However, researches provide us by some important information. In 1987, some investigator recorded the presence of a mild form of RVF disease circulating as a sub-clinical infection among animals in Sharqyia Governorate as atypical RVF epidemic or epizootic [18]. Other study was described the pathological picture of RVF on suspected heifers from Friesian dairy farm with a history of abortion in 1989 [19]. Meanwhile, in a study of the prevalence of antibodies to RVFV in 915 persons in the Nile river delta of Egypt, the IgG antibody to RVF virus was detected in 15% of specimens in 1993 [20]. AHRI mentioned in an official report about RVF situation in Egypt that RVFV was isolated from limited numbers of native animals in Keina governorate of Upper Egypt in 1996 and in Damitta governorate of Nile Delta of Egypt in 2003 (AHRI, personal communication). In like manner, NAMRU-3 has been reported that it was played a key role in the outbreak investigations in Egypt in 1996 and 2003 [21].

Also, during year 2000, 4,161 serum samples were collected from different Governorates 1-6 months post vaccination with inactivated vaccine, a total of 977 cattle, 743 buffaloes, 1,127 sheep and 1,314 goats. The infectivity for each species ranged from 5.78, 9.58, 12.28 and 13.59 for goat, sheep, cattle, and buffalo respectively. The infectivity percentage in some animals indicates that the virus still circulating in the presence of the insect vector. The susceptibility of different species (animals have neither IgM nor IgG) were 13.11%. Sheep showed low susceptibility by 9.41%, indicating the concentration of vaccination program on sheep. Some animals were escaped vaccination and others showed immune-suppressive diseases [22].

### Largest RVFV outbreaks in Egypt

#### The 1st outbreak 1977-1978

The first outbreak of RVF in Egypt was recorded at Sharqiya Governorate in October 1977. The outbreak first appeared in man as an acute, febrile, dengue-like illness in Sharqiya Governorate in Egypt. Then, RVFV was isolated from man at the virus Research Center, Agousa district in Cairo and characterized as an Arbovirus. This isolate (isolate no. 41) was confirmed as RVFV at the WHO collaborating center for Reference and Research on Arbovirus Disease at Yale Arbovirus Research Unit (YARU), USA [17,18]. However, the veterinarians who were sharing in controlling this early outbreak in 1977, stated that RVF entered Egypt for the first time by the Egyptian troops who came back after peace keeping mission in the Congo and the outbreak in animals and human began at the same place of these infected troops in Belbies city, Sharqiya Governorate [Safi Al-Din Sakr, GOVS, Sharqiya branch, personal communication] [19].

As a matter of fact, during periods in which human epidemics arise they are preceded by epizootics in livestock. These livestock epizootics serve as an amplification step in the spread of the virus. This outbreak indicated that man can serve as RVFV amplified subject. This can be achieved by the circulation of the virus between insect vector and other vertebrates in the human dwellings. Additionally, there are some controversies in recording for this important outbreak. Some reports stated that RVFV probably introduced in Egypt by importing infected sheep and camels [20,21]. Other investigations mentioned that during the epidemic of RVF that occurred in Egypt and other areas of North Africa in 1977, the virus was isolated from various species of domestic animals and rats (Rattus rattus frugivorus) as well as man. Also, the highest numbers of RVFV isolates were obtained from sheep; only one isolate was recovered from each of the other species tested, viz. cow, camel, goat, horse, and rat.

The human losses in this outbreak were massive. The lack of previous medical experience about RVF patients and the insufficient health education programs considered principal factors in this concern. Likewise, the host innate immune response plays a major role in RVF host resistance. Therefore, hepatic affections are predispose to the severity of the illness in Egyptian farmers who suffering Schistosomiasis, HCV, HBV, HCC and Cirrhosis. As well as, infants, very young children and immune compromised patients are more liable to RVFV.

#### The 2nd outbreak 1993

The source of RVFV in this outbreak was probably due to natural infection or vaccinal strains. It was recorded that the Egyptian RVFV isolates group contains strains isolated in 1977 and 1993 epidemics, was appeared in the phylogeny as sister groups. This finding was suggesting that either the virus remained endemic between the two outbreaks or have been reintroduced in 1993 from the same source (probably Sudan) as in 1977 [22]. However, some studies were suggested that the virus was reintroduced through an incompletely inactivated RVF veterinary vaccine [23]. However, this outbreak probably was due to post-vaccinal reactions with reversion to virulence of the vaccinal strain. During 1993, the AHRI mission was reporting RVF in governmental farms, located in North of Egypt (Damitta Governorate). These farms were vaccinated by RVF attenuated vaccine 20 days prior to the onset of the disease [the author was a member of this mission]. The pregnant cows, adults, and calves were suffering severe illness, mortalities and a storm of abortion following vaccination with the live attenuated RVF vaccine (Smithburn strain). This live vaccine was imported by the Egyptian General Organization for Veterinary Services (GOVS) from the South Africa (Veterinary Research Institute, Onderstepoort, 01100 South Africa, Batch No.G119 in terms of Act 36 of 1947). The exact reason for importing this vaccine is unknown either the real date or place of first vaccination or field trials in Egypt.

#### The 3rd outbreak 1994

During 1994, RVFV was isolated from the infected cattle and sheep in Behera and Kafr el Sheikh Governorates The serological investigations revealed 31.65% and 57.1% prevalence of virus in 139 cattle and 84 sheep, respectively. The locally produced live attenuated RVF vaccine, Smithburn strain, was officially applied for use in Egypt the same year (Batch No. 1-1994) [24]. This outbreak showed the failure of the locally applied RVF vaccination program with both the imported and the locally produced live attenuated vaccine (Smithburn strain).

#### The 4th outbreak 1997

At the beginning of the summer of 1997, a high incidence of abortion was observed among pregnant sheep and cattle with high mortalities in young lambs and calves during the summer of 1997 in Upper Egypt. The disease was more severe in sheep than in cattle and in young animals than in adults. The abortion rate in pregnant ewes was approximately 60%-70% and in pregnant cows approximately 30%-40%. A mortality rate of approximately 50%-60% was recorded in young lambs, 25%-35% in adult sheep, 25%-30% in calves and 10%-20% in adult cattle. This study reveals massive infections and indicated the absence of an effective traceability system in Egypt [25].

Also, during 1997, a concurrent infection of Theileriosis and RVFV among calves and cattle in dairy farm were reported. The affected farm which located in Assiut governorate, Upper Egypt was containing both native cattle (110) and Holstein Friesian (104) [26].

These findings reveal the failure of vaccination programs by the live RVF vaccines. Despite, RVFV is characterized by the presence of inter-epizootic periods of 10-15 years, this outbreak occurred a 3 years only after the last epizootic in 1994. Additionally, RVFV infections under natural conditions are usually subside and the herd acquired solid immunity until a naïve herd present in sufficient numbers. This unusual behavior of the virus was probably a post-vaccinal reaction of the live vacinal strains (Smithburn strain) beside the continuous importation of infected ruminants, especially camels from enzootic areas in Africa [25].

#### The 5th outbreak 2003

The biggest market of livestock in Egypt located in Kafr el Sheikh Governorate, about 150 km north of Cairo, where animals are collected from all over the country. Such environment predisposed to another RVFV outbreak during year 2003. This outbreak was encountered in different localities of Egypt [27]. The RVF disease in human probably was owned to the direct contact with infected animals, or through infected droplets during the slaughtering of sick cattle, or through bites of mosquitoes fed on infected blood. The farmers are usually slaughtering the sick animals, resulting in the spread of the highly amplified virulent RVFV in the surroundings. In similar manner, students at a South African veterinary college, several veterinarians and veterinary staff were infected after handling and performing necropsies on animals that were only later identified as infected with RVF virus [28,29].

Thus human infection seems inevitable during RVF epizootics. RVFV amplification cycles in livestock frequently precede human cases by 34 weeks, and play a critical role in the early stages of an outbreak. The highly viraemic (virus circulated in blood) animals serve as an excellent source of direct contamination of humans, as well as a blood meal source for mosquitoes which can transmit the virus to humans [30]. The same finding was obtained by the analysis of livestock and human data in the RVFV outbreak in Kenya (2006 and 2007) which suggests livestock infections would be occurred before virus detection in humans [31].

In spite of the severe epidemic, the Egyptian Ministry of Agriculture refuses to announce this epizootic till the present time. However, as of 28 August 2003, WHO received reports of 45 cases of Rift Valley fever (RVF) including 17 deaths in Seedy Salim district, a remote rural area in Kafr Al-Sheikh governorate, about150 km north of Cairo. All cases are Egyptian farmers. Laboratory testing carried out at the Naval Medical Research Unit No.3 (NAMRU-3), Cairo, has confirmed the diagnosis of RVF in clinical samples. A gradual increase in the number of suspected cases of RVF in Seedy Salim district has been reported as a result of active surveillance [32].

The RVFV was isolated by AHRI from a veterinary samples collected from Damitta Governorate during year 2003 (AHRI, personal communication). Recently the scientific data regarding this outbreak was referred to the spread of RVF all over the country. In June, 2003, Egypt's hospital-based electronic disease surveillance system began to record increased cases of acute febrile illness from governorates in the Nile Delta. In response to a request for assistance from the Egyptian Ministry of Health and the World Health Organization (WHO), the U.S. Naval Medical Research Unit No. 3 (NAMRU-3) provided assistance in identifying the cause and extent of this outbreak. Testing of human clinical samples (n = 375) from nine governorates in Egypt identified 29 cases of RVF viraemia that spanned the period of June to October, and a particular focus of disease in Kafr el Sheikh governorate (7.7% RVF infection rate). Veterinary samples (n = 101) collected during this time in Kafr el Sheikh Governorate (Egypt) and screened by immunoassay for RVFV-specific IgM identified probable recent infections in cattle (10.4%) and sheep (5%). Entomologic investigations identified three isolates of RVF virus (RVFV) from 297 tested pools of female mosquitoes and all three RVFV isolates came from Cx. antennatus (Becker). This is the first time that Cx. antennatus has been found naturally infected with RVFV in Egypt [27] Table 1).

**Table 1:** RVFV outbreaks in Egypt

Year	Vaccines	Characteristics
**1977-1978**	No vaccination	RVFV introduced from Africa by infected persons back from Africa & animals from the Sudan, probably viraemic camels. Showed historic massive human losses. Led to the establishment of the endemic state. RVFV isolation from infected humans and animals

**1993-1994**	Live attenuated (Smithburn strain), Used during the outbreak	Epizootic and Epidemic. Probably natural infections, beside Vaccinal strains. Source of virus from the Sudan by infected ruminants. Infections encountered in all Egyptian Governorates.

**1996-1997**	Live attenuated (Smithburn strain), Used during the outbreak	Epizootic and Epidemic. Source of data: literatures, OIE, NAMRU-3 Cairo. Showed human infections among some farmers and veterinarians. Indicate the failure of the applied vaccination programs. Showed failure of general authority to face the real situation. Questionable traceability of the GOVS, *AHRI, *VSVRI. Importation of infected animals without proper laboratory tests.

**2003**	Live attenuated (Smithburn strain), Used during the outbreak	Epizootic and Epidemic. Encountered in Nile Delta & Upper Egypt Governorates. Source of data: WHO, NAMRU-3 Cairo, Literatures. Many human losses among farmers. Massive losses in animals. Indicate the failure of the applied vaccination programs. Indicate failure of general authority to face the real situation. Questionable traceability of the GOVS, *AHRI, *VSVRI

*Animal Health Research Institute (AHRI). *Veterinary Serum &Vaccine Research Institute (VSVRI)

## The role of camels in RVFV transmission

Camels are multipurpose domestic animals used for meat, hair and hide production beside transportation. Also, camels play certain role in the continuous introduction of RVFV in Egypt. On the contrary to the OIE rules, camels enter Egypt officially without any virological investigation, and even without maintaining for sufficient period in the quarantine facilities. The GOVS consider the long distance the animals spent walking is enough for judging that they are free from the viral infections. In reality, this is not matched the OIE rules for importing live animals.

The continuous importation of viraemic ruminants, especially camels, from the Sudan was the main source of infection in RVF and FMD outbreaks in Egypt [25]. Furthermore, RVFV infections are due to live animal's importation. However, the importation from Africa consider of high risk due to the presence of trans-boundary animal diseases (TADs) such as Rift Valley Fever and Foot and Mouth Disease (FMD), with the absence of an effective traceability system that acts as proxy for quality assurance [33].

Serologic evidence of RVF in camels is frequently reported. Widespread abortion waves in camels were observed during RVF outbreaks in Kenya and Egypt. Camels are suspected of playing a major role in the spread of RVF from northern Sudan to southern Egypt in 1977. Also, RVF virus was previously isolated from blood samples from healthy, naturally infected camels in Egypt and Sudan. Although, RVFV susceptibility varied from species to another, some virulent strains could change the classic picture of the epizootics. This was observed during September-October 2010, when an unprecedented outbreak of Rift Valley fever was reported in the northern Sahelian region of Mauritania after exceptionally heavy rainfall. Camels probably played a central role in the local amplification of the virus. During this outbreak, two clinical forms were observed in camels: Peracute form, with sudden death in less than 24 h; and acute form with fever, various systemic lesions and abortions. When hemorrhagic signs developed, death usually occurred within a few days in manner resembles infections in the most susceptible species (sheep) [34].

## RVF vaccines used in Egypt

Currently, there are no licensed vaccines for RVF that are both safe and efficacious for human use. The following vaccines are for veterinary use only: 1- Live attenuated Smithburn strains, produced by VSVRI, 2- Formalin inactivated, alum adjuvanted, (Menya/Sheep/258) produced by VACSERA, 3- Binary ethylenamine inactivated (ZH501 RVF strain) and Alum adjuvanted, which produced by VSVRI.

Control of RVF in Egypt depends mainly on periodical vector control and vaccination of susceptible animals with binary inactivated RVF vaccine. Smithburn's neurotropic attenuated RVFV strain is a 104th intracerebral passage in baby mice followed by two passages in baby hamster kidney cell line. It was 10 ^7.5 ^TCID _50/ml. _However, questions exist concerning the abortogenicity and teratogenicity of the preparation as well as its phenotypic stability [35].

The description of the live attenuated RVF vaccine (smithburn strain) which produced by the Egyptian VSVRI stated the following; a- Live attenuated freeze dried tissue culture Rift Valley Fever virus vaccine (smithburn strain). b- The vaccine contains 104 TCID 50/ml. RVFV. c- The virus grows on tissue culture cell line. d- It is indicated for the protection of sheep and goat against Rift Valley Fever disease. e- The vaccine not used for pregnant animals as the vaccine may cause abortion or embryo abnormalities. f- The dosage and method of use; 1- the content of the vaccine vial dissolved in 100 ml. 2- sterile distilled water or physiological saline.3- each animal can be vaccinated by 1 ml s/c. 4- Sheep and goat should be vaccinated at 4 months of age. Remarks: 1- Avoid exposure of the vaccine to direct sunlight and heat. 2-Clinically ill animals should not be vaccinated.3- Do not vaccinate animals during breeding season of mosquitoes. 4- Animals used for human consumption should not be slaughtered within 21 days after vaccination. 5- Used syringes, needles and remaining vaccine in bottles should be disposed hygienically. The vaccine should be stored at - 20°C. Expire date is 2 years from the date of manufacture [36].

In the light of this description we can realize how much this vaccine is unsafe. It is for use in sheep and goats only. As a fact, the mosquito's breeding season in Egypt is 12 month per year because of the warm winter and the other ecological and environmental factors. However, the safety requirements of using it in the absence of mosquito, as mosquitos present anywhere and anytime of the year make it impractical for general use. The vaccinated animals shouldn't be slaughtered within 21 days after vaccination, because of viraemia. In experimental study performed by VSVRI and NARU-3, a number of 318 cows and 115 buffaloes were vaccinated with the locally prepared RVF Smithburn vaccine, of which, 100 cows and 20 buffaloes were pregnant. Twenty eight pregnant cows were aborted within 72 days post-vaccination. Buffaloes didn't abort. RVFV was isolated from one aborted fetus. Moreover, the antibody response to vaccination with local Smithburn strain had occured in some, but not all the cows and buffaloes. Virus isolation from the fetus suggests in utero transmission of the used vaccinal strain, which resulted in high abortions in cows [37].

Field study was carried out in Alexandria, Egypt, to assess the use of locally produced inactivated and attenuated Rift Valley fever (RVF) vaccines on lambs and calves. The study recommends the usage of RVF inactivated vaccine because it was safe and gave results as live attenuated vaccine especially with a poster dose after 5 months of the first vaccination [38].

## Discussion

RVFV is endemic in Africa and Arabian Peninsula [39]. This virus is classified as a high-consequence pathogen with the potential for international spread (List A) by the World Organization for Animal Health (Office International des Epizooties) [40]. The disease is fatal in man with unhealthy liver and immunocompromised patients are more liable to the disease. RVF control in animals through the use of safe and effective vaccine will prevent the human disease by breaking the amplification cycle [41].

RVF virus amplification cycles in livestock frequently precede human cases by 34 weeks, and play a critical role in the early stages of an outbreak. These highly viraemic (virus circulated in blood) animals serve as an excellent source of direct contamination of humans, as well as a blood meal source for mosquitoes which can transmit the virus to humans [42].

In the present time, Egypt is importing Camels, cattle, and small ruminants from the horn of African & the Sudan which are not vaccinated against RVFV. However, in case of importation from countries which vaccinate against RVFV, the imported animals were with no date of vaccination and the unvaccinated control contacts were unavailable for further veterinary investigations. The regulatory plan for livestock importation was to slaughter these animals upon their arrival for human consumption. Some of these animals usually escaped slaughtering for breeding which led to the spread of new strains of FMD and introduction of RVFV from enzootic areas. However, the vaccination programs by killed vaccines only which applied by Ministry of Agriculture have been limiting to great extent the possibilities of RVFV outbreaks in Egypt.

Control of RVF disease in Egypt depends mainly on vaccination of cattle, sheep and goats. Two types of formalin inactivated RVF vaccines were produced in Egypt; first produced by VACSERA company which is formalin inactivated & alum adjuvanted (Menya/Sheep/258), and the second produced by the Veterinary Serum and Vaccine Research Institute (VSVRI) which is binary ethylenimine (BEI) inactivated & alum adjuvanted (ZH501 strain) [43,44].

The live attenuated RVF Smithburn vaccine also referred as Smithburn neurotropic strain or SNS. The Smithburn neurotropic strain of RVF virus was derived from the virulent Entebbe strain by numerous serial intracerebral passages in mice [45]. This live vaccine was imported from South Africa and subsequently produced by VSVRI according to protocol of WHO/FAO (1983) [46].

In the light of the use of live attenuated RVF vaccine (smithburn strain) for animals consider of high risk as it causes abortion in pregnant animals, teratogenic effects and the reversion to be virulent is possible. The VSVRI stopped producing the live attenuated RVF vaccine (Smithburn strain) as the Rift Valley fever department, was reported the possibility of the vaccinal strain reversion to virulence state [37,44,47,48].

As has been noted in a research studies performed in VSVRI, the RVF live vaccine adverse effects were illustrated [37,47,48]. They mentioned that a locally inactivated RVF vaccine was produced for vaccination of cattle and sheep. The inactivated vaccine was still used until the appearance of the outbreak in Egypt in 1993. The general authority uses the imported RVF attenuated vaccine (smithburn strain) to overcome this problem. Yet, a locally attenuated RVF vaccine was produced from Smithburn strain in 1994 (modified live virus vaccine MLVV). This attenuated vaccine was used together with the inactivated one for vaccination of farm animals, but the live virus vaccine may cause abortion, and its potential for reversion to virulence has not been adequately investigated [37,47].

They added, WHO/FAO meeting (1983) warranted the use of such attenuated vaccine as there are major practical and theoretical reasons for not using a live vaccine in non-endemic RVF zones and in some endemic zones. Lambs immunized at less than 6 weeks of age have a low incidence of encephalitis and ewes immunized shortly before giving birth may produce lambs with encephalitis. In addition, MLVV induces abortion in a small percentage of pregnant ewes and may cause fetal abnormalities. Also, the FAO meeting added that there is a theoretical objection to use of a live attenuated vaccine, since such vaccine might revert to virulence for cattle, sheep or man. Moreover, the live vaccine has the capacity to cause viraemia in vaccinated sheep, and therefore, arthropods feeding on these animals may become infected and transmit the disease to domestic animals or man. They conclude that despite the disadvantages of the Smithburn RVFV attenuated vaccine, their study explores another disadvantage as the RVFV appeared in bull's semen 1 week after their vaccination with the attenuated RVF vaccine with an average titre of 1 ELISA unit. Also, RVFV was detected till the third week post-vaccination, and the semen quality was severely affected. Thus, vaccination of bulls with this attenuated RVF vaccine leads to dissemination of RVFV via semen as if they were infected, and the use of these bulls for insemination might be of danger, somehow to these bulls as well as the inseminated cows [47].

Vaccination with live attenuated vaccine was applied in Egypt at intermittent periods, since the 1977 outbreak, and killed vaccine was used for pregnant and young animals [44,47]. The Egyptian GOVS in 2008 mentioned that RVF live vaccine (Smithburn strain) don't used in Arab Republic of Egypt in the present time. Consequently, the RVF vaccination programs in Egypt are performed by the killed vaccines only [44].

MP12 was invented by serial mutagenesis which was undertaken with the Egyptian ZH501 and ZH548 strains of RVF virus. Strain ZH501, which was received as a first-passage virus grown in foetal rhesus monkey lung (FRL) cells, originally came from the serum of a fatal haemorrhagic case of human RVF that occurred in the Sharqiya Governorate during the Egyptian outbreak of RVF in 1977. Strain ZH548, which was received after two passages in suckling mice and one in FRL cells, was recovered originally from the serum of an uncomplicated human febrile case of RVF that occurred in the same area of Egypt. A total of 18 serial mutageneses were undertaken with ZH501, and 16 with ZH548. Mutagenesis consisted of growth of virus in the presence of 5-fluorouracil (5FU) [49].

However, some studies showed that MP12 strain replicated in and was transmitted by female C. pipens after intrathoracic inoculation [50]. Also, It was reported that MP12 was evaluated in a group of 50 sheep at various stages of pregnancy was inoculated with the virus and the pregnancies followed to term. There were two abortions and 14% of the lambs produced by vaccinated ewes showed teratogenic effects, the most prevalent being spinal hypoplasia, hydranencephaly, brachygnathia inferior and arthrygryposis. The fetal malformations of the central nervous and musculoskeletal systems were mostly consistent with those observed in sheep vaccinated with the attenuated Smithburn RVF strain [51].

The Egyptian General Organization of Veterinary Services (GOVS) policy of using inactivated vaccine (only) is a candidate to change. A recent project of the GOVS of Egypt is working to test the attenuated live vaccine MP-12 for possibility of its production and usage in Egypt. This project is collaboration with NAMRU-3. They reports on a project that is testing a vaccine developed by the U.S. Army, called MP-12, on Egyptian domestic animals. This project, collaboration with the Veterinary Serum and Vaccine Research Institute of the Egyptian Ministry of Agriculture and the U.S. Department of Agriculture is studying how well this vaccine works. It is one of the most promising vaccines to date, and if effective, could be tested in the field in Egypt [21].

In addition to the disadvantages of using live vaccines, some literatures also revealed that RVF MP12 strain is not safe for using in endemic countries as Egypt. It was mentioned that to assess the genetic variability of Rift Valley fever virus (RVFV), several isolates from diverse localities of Africa were investigated by means of reverse transcription-PCR followed by direct sequencing of a region of the small (S), medium (M), and large (L) genomic segments. Phylogenetic analysis showed the existence of three major lineages corresponding to geographic variants from West Africa, Egypt, and Central-East Africa. However, incongruence detected between the L, M, and S phylogenies suggested that genetic exchange via reassortment occurred between strains from different lineages. This hypothesis, depicted by parallel phylogenies, was further confirmed by statistical tests. These findings, which strongly suggest exchanges between strains from areas of endemicity in West and East Africa, strengthen the potential existence of a sylvatic cycle in the tropical rain forest. This also emphasizes the risk of generating uncontrolled chimeric viruses by using live attenuated vaccines in areas of endemicity [52].

The mutations responsible for attenuation of RVFV have been examined by analysis of reassortant viruses generated between the vaccine strain and a wild RVFV strain isolated in Senegal. Their findings indicate that genetic reassortment with wild-type viruses during a vaccination program in endemic areas would also be expected to yield attenuated variants [53].

New strains of live attenuated RVFV are created by using reverse genetics that is missing one or more viral virulence factors, which facilitating the studies of viral pathogenesis and the development of specifically attenuated vaccine strains. This system has been used to generate viruses that are missing the NSs protein, the NSm protein, or both. In addition, unlike the currently available attenuated strains, the ΔNSm/ΔNSs vaccine meets the DIVA requirement by virtue of the missing NSs protein [13].

Unfortunately, in spite of vaccination is frequently believed to be an innocuous procedure, it is important to recognize that vaccines can cause adverse reactions, as vaccination is a serious medical act. However, recombination between live vaccinal strains and virulent strains is possible under specific conditions, and may result in reversion to virulence of recombinant DNA (rDNA) vaccines or conventional vaccines. Furthermore, vaccines marked by the deletion of a gene can regain the deleted gene, with dramatic consequences for eradication program [54].

## Conclusions

The importation of animals infected with RVF from the Sudan and failure of the locally applied RVF vaccination program were the main causes of the reoccurrence of RVF epizootics in Egypt. Continued outbreaks of RVF among domestic ruminants, in 1977, 1978 and 1993, 1994, 1996, 1997, and 2003 indicate that the virus has become enzootic in Egypt. To control the disease in Egypt, some investigators suggest the following measures: - prevention of introduction of ruminants infected with RVF, especially camels, from northern Sudan into southern Egypt (Aswan Province) - avoiding importation of ruminants from countries where RVF is enzootic - annual vaccination of all ruminants (camels, cattle, buffaloes, sheep and goats) with an effective RVF vaccine - trials for control of insect vectors - the intensive broadcast of educational television programs to inform farmers and owners about the importance of vaccination programs [25].

Two live attenuated viruses have been tested in various animals, a mutagen-attenuated strain (MP12) and the live attenuated Smithburn strain. The results of these studies with live vaccines are varied, in some instances showing no clinical illness and the development of neutralizing antibody titers as well as protection from challenge, and in other studies showing the viruses to be abortogenic and teratogenic. Therefore neither of these virus strains appears to be an ideal candidate for a vaccine strain because of their questionable safety profiles, in addition to their lack of DIVA capability [13].

Under those circumstances, it could be concluded that the use of inactivated RVFV vaccine is safe and effective in controlling RVFV especially when the vaccination programs is strictly applied and cover all domestic animals in Egypt.

## Competing interests

The author declares that they have no competing interests.

## Authors' contributions

SAK conceived of the study, and participated in its design and coordination. The author read and approved the final manuscript.
